# Phenotypic Data from Inbred Parents Can Improve Genomic Prediction in Pearl Millet Hybrids

**DOI:** 10.1534/g3.118.200242

**Published:** 2018-05-24

**Authors:** Zhikai Liang, Shashi K. Gupta, Cheng-Ting Yeh, Yang Zhang, Daniel W. Ngu, Ramesh Kumar, Hemant T. Patil, Kanulal D. Mungra, Dev Vart Yadav, Abhishek Rathore, Rakesh K. Srivastava, Rajeev Gupta, Jinliang Yang, Rajeev K. Varshney, Patrick S. Schnable, James C. Schnable

**Affiliations:** * University of Nebraska-Lincoln, Lincoln, NE; † International Crops Research Institute for the Semi-Arid Tropics (ICRISAT), Hyderabad, Telangana State, India; ‡ Iowa State University, Ames, IA; § Chaudhary Charan Singh Haryana Agricultural University, Hisar, Haryana, India; ** Mahatma Phule Krishi Vidyapeeth, Dhule, Maharashtra, India; †† Junagadh Agricultural University, Jamnagar, Gujarat, India

**Keywords:** pearl millet, Genomic Selection, hybrid breeding, genotyping, GenPred, Shared Data Resources

## Abstract

Pearl millet is a non-model grain and fodder crop adapted to extremely hot and dry environments globally. In India, a great deal of public and private sectors’ investment has focused on developing pearl millet single cross hybrids based on the cytoplasmic-genetic male sterility (CMS) system, while in Africa most pearl millet production relies on open pollinated varieties. Pearl millet lines were phenotyped for both the inbred parents and hybrids stage. Many breeding efforts focus on phenotypic selection of inbred parents to generate improved parental lines and hybrids. This study evaluated two genotyping techniques and four genomic selection schemes in pearl millet. Despite the fact that 6× more sequencing data were generated per sample for RAD-seq than for tGBS, tGBS yielded more than 2× as many informative SNPs (defined as those having MAF > 0.05) than RAD-seq. A genomic prediction scheme utilizing only data from hybrids generated prediction accuracies (median) ranging from 0.73-0.74 (1000-grain weight), 0.87-0.89 (days to flowering time), 0.48-0.51 (grain yield) and 0.72-0.73 (plant height). For traits with little to no heterosis, hybrid only and hybrid/inbred prediction schemes performed almost equivalently. For traits with significant mid-parent heterosis, the direct inclusion of phenotypic data from inbred lines significantly (*P* < 0.05) reduced prediction accuracy when all lines were analyzed together. However, when inbreds and hybrid trait values were both scored relative to the mean trait values for the respective populations, the inclusion of inbred phenotypic datasets moderately improved genomic predictions of the hybrid genomic estimated breeding values. Here we show that modern approaches to genotyping by sequencing can enable genomic selection in pearl millet. While historical pearl millet breeding records include a wealth of phenotypic data from inbred lines, we demonstrate that the naive incorporation of this data into a hybrid breeding program can reduce prediction accuracy, while controlling for the effects of heterosis *per se* allowed inbred genotype and trait data to improve the accuracy of genomic estimated breeding values for pearl millet hybrids.

Pearl millet [*Cenchrus americanus* (L.) Morrone; Syn. *Pennisetum glaucum* (L.) R. Br.] is able to grow on infertile and marginal soils under limiting soil moisture conditions and high soil temperatures. It is a climate resilient species, and is one of the most widely grown millets globally Ramya *et al.* (2017). Pearl millet can thrive in arid environments, and successfully set seed at temperatures above 40°, which would kill the pollen/stigmas of many other grain crops Gupta *et al.* (2015). Pearl millet can also tolerate infertile and marginal soils, limited soil moisture, and high soil temperatures. While most pearl millet production in Africa utilizes open pollinated varieties, Indian pearl millet production now makes extensive use of hybrid seed generated using three line cytoplasmic-genetic male sterility systems (CMS) Hanna (1989). Three line CMS systems employ female lines which carry male sterile cytoplasm and non-restoring nuclear gene(s) (A-lines), maintainer lines carry an identical nuclear genome to each A-line in a compatible fertile cytoplasm, resulting in male fertile plants (B-lines) and are able to maintain the male sterility of A-line, and pollinator/male lines which carry dominant nuclear restorer of fertility gene(s) (R-lines).

Plant breeding for hybrid crops requires generating and testing large numbers of hybrids under different field conditions. Performing crosses to generate F1 hybrids is a labor intensive process. Top-crossing between B- and R-lines can reduce the amount of labor required per cross, but only in crossing schemes where many female lines are being crossed to one or a few male lines. Evaluating each new hybrid across field trials with several environments also requires significant time and resources. As a result, methods for selecting parental inbred lines and determining which crosses are likely to yield the best hybrids is a critical part of crop improvement. In pearl millet, a widespread approach has been used to evaluate the phenotypes of new potential inbred parents as a first pass screen of their potential in hybrid breeding programs.

Traditionally, mid-parental values have been a common way to predict performance of hybrids on the basis of inbred values, combined with estimates of General Combining Ability (GCA) in cases where phenotypes cannot be scored in parental lines or individuals directly Gowda *et al.* (2013); Xing *et al.* (2014); Mühleisen *et al.* (2015). However, for traits where significant heterosis exists, the phenotypes of hybrids can vary significantly from what would be predicted through the use of mid-parent values and estimated GCA. In these cases, it can be necessary to estimate Specific Combining Ability (SCA) values for each potential cross. The incorporation of genetic markers can improve the accuracy with which both GCA and SCA can be predicted by enabling the sharing of data across multiple tested lines carrying common haplotypes Schrag *et al.* (2007). When applied to sets of genetic markers across the whole genome, this process is referred to as genomic prediction (GP), which can be used to implement breeding programs based on estimated breeding values from genome wide sets of markers, a process know as genomic selection (GS).

Approximately 90-100 pearl millet hybrids are currently cultivated on about 5 million hectares in India Yadav *et al.* (2016). Both public and private sector organizations, including 30-40 seed companies, perform thousands of test-crosses each year. Resulting hybrids are then evaluated over multiple years and multiple locations to identify small numbers of new hybrids with superior performance which can be marketed/released for cultivation. The high investment of time and resources into initial hybrid evaluation would benefit significantly from the use of GP/GS to exclude many potential test crosses which can be discarded as unlikely to outperform existing hybrids prior to field evaluation, reducing the vast number of crosses which must be performed and evaluated.

The use of GP/GS to obtain estimated breeding values have been widely evaluated and employed in inbreeding crops such as wheat Poland *et al.* (2012), barley Zhong *et al.* (2009), rice Spindel *et al.* (2015). In crops where production is based upon hybrids, genomic prediction for single-cross hybrid performance are only starting to appear in the public sector literature Technow *et al.* (2014); Kadam *et al.* (2016), although genomic predictions for hybrid performance across populations all crossed to a single common tester are more common Windhausen *et al.* (2012); Albrecht *et al.* (2014). Pearl millet presents an intriguing opportunity in that both hybrid and open pollinated production systems are widely employed, and phenotypic data are thus available from both hybrids and inbred R- and B- lines. A-lines, being male sterile, do not produce grain when grown in isolation.

Here we evaluated two potential genotyping strategies – RAD-seq Miller *et al.* (2007) and tGBS Ott *et al.* (2017) to characterize a set of inbred pearl millet lines developed by ICRISAT in Hyderabad, India, and then evaluated the utility of GP/GS to predict optimal hybrid combinations among possible combinations of these inbreds using a scheme trained using phenotypic data collected from hybrid trials alone, inbred trials alone, or both.

## Materials and Methods

### Field Traits

Field trials were conducted at four locations in India, spanning two agro-ecological zones (A- and B- zone, having rainfall of >400mm/annum) of pearl millet cultivation. The Hisar and Jamnagar sites fall within the A zone of pearl millet cultivation in northwest India, while Dhule and Patancheru are located in the B zone of pearl millet cultivation in southern (peninsular) India Gupta *et al.* (2013). While pearl millet is also grown in the A1 zone - highly drought prone areas with less than 400 mm of rainfall per year - the majority of hybrid pearl millet is confined to the A and B agroecological zones. Data were collected from 320 hybrids and 37 inbreds at field trials in four locations in 2015 in India (Dhule: N20.90°,E74.77°; Patancheru: N17.53°,E78.27°; Jamnagar: N22.47°,E70.06° and Hisar: N29.10°,E75.46°). In CMS system, A-lines are sterile and hence will not produce grain when grown in isolation. Therefore, genotyping and phenotyping were conducted on non-sterile B-lines carrying the same nuclear genome as A-lines in a compatible cytoplasm, rendering them male fertile. Lines in plots were grown in an alpha lattice design with two replicates and 28 15-plot blocks in each location. Each block included two common control lines/hybrids (ICMH 356 and 9444) and 13 experimental lines. Hybrid plots were randomly assigned to the first 25 blocks of each replicate, and inbred plots were randomly assigned to the last three blocks of each replicate (Experimental design, plot distribution and recorded phenotypes was provided in FigShare https://figshare.com/articles/pearl_millet_genomic_selection_field_layout/5969230).

### Phenotype measurement

Phenotypic traits scored include days to 50% flowering (days), plant height (centimeters), grain yield (kilograms/hectare), and 1000-grain weight (grams). The criteria used to measure each of these four traits were as follows. 1) Plant height (centimeters): Plant height for a given plant was measured from where the main stem meets to soil to the tip of the panicle of the primary tiller at the time of harvest. For each plot, five random plants were randomly selected for height measurements and the mean value of these five measurements was reported; 2) Days to 50% flowering (days): Days to flowering was measured as the time between the planting date and the date at which at least 50% of plants within a given plot exhibited the initiation of stigma emergence on the panicle of their primary tiller; 3) 1000 seed weight (grams): 200 seeds were counted out from the pooled grain collected from a given research plot, weighed, and multiplied by a factor of 5 to determine 1000 seed weight (grams); 4) Grain yield (kilograms/hectare): For each entry, all panicles within a given a plot were harvested at physiological maturity, and these panicles were sun dried for 10 to 15 days and then threshed for grain yield. Planting density and plot size varied across locations, but for each location the total yield was multiplied by the number of plots per hectare to estimate the final yield per hectare.

### DNA extraction and library construction

Thirty to thirty five seeds from each inbred line were sown in a four inch pot in a darkroom at ICRISAT°s Patancheru. The pots were maintained at a temperature between 18° and 25°. Etiolated leaf tissues were harvested eight days after planting. Pooled leaf tissue from 20 to 25 seedlings per line was collected for DNA extraction. DNA was extracted using a modified DNA extraction method described by Mace *et al.* (2003). The DNA was stained by 5 ng/µl of ethidium bromide and checked using 0.8% (w/v) agarose gel electrophoresis in Tris-acetate-EDTA (TAE) buffer for 1 h at 90 V with visualization under ultraviolet (UV) light. tGBS sequencing libraries for 192 B-lines and 192 R-lines were prepared following the protocol outlined in Ott *et al.* (2017). RAD-seq libraries for a set of inbreds including all but 12 of the lines genotyped using tGBS were constructed as described in Varshney *et al.* (2017). RAD-seq libraries were sequenced using an HiSeq 2000 and tGBS libraries were sequenced using an Ion Proton (Table 1).

**Table 1:** Comparison between RAD-seq and tGBS genotyping technologies

	RAD-seq	tGBS
Total number of samples genotyped	372	384
Sequencing platform	Paired-end	Single-end
	Illumina HiSeq 2000	Ion Proton
Average (Median) Reads/Sample after QC	12,221,976 (12,097,256)	1,793,300 (1,365,265)
Average (Median) Sequence/Sample after QC	965,295,176 (955,561,340)	195,057,311 (146,026,776)
Average (Median) missing rate / SNP	41.39% (41.67%)	58.65% (63.02%)
Average (Median) Proportion Het Calls / SNP before imputation	2.05% (0.42%)	4.12% (3.82%)
Average (Median) Proportion Het Calls / SNP after imputation	1.63% (0.53%)	4.72% (2.86%)
Average (Median) MAF / SNP before imputation	1.89% (1.18%)	11.69% (5.43%)
Average (Median) MAF / SNP after imputation	1.24% (0.67%)	10.37% (3.26%)
Total SNPs	649,067	73,291
SNPs with MAF >0.05 after imputation	15,306	32,463

### SNP calling and filtering

Raw sequence data obtained from both genotyping strategies was analyzed using the same analytical pipeline to enable accurate comparisons between the two. Raw reads were aligned to the pearl millet reference genome (v1.1 Varshney *et al.* (2017)) using default settings of GSNAP Wu and Nacu (2010).

After alignment, SNPs were called using the software package 123SNP Yu *et al.* (2012) ignoring the first and end 3bp of aligned reads. After ignoring the first and last 3 bp of each read, polymorphic sites were determined using the following criteria: 1) 5 aligned reads covering the position in the genome; 2) PHRED quality greater than 20. Genotype calls for individual samples were determined in the following fashion. The genotype for a given SNP marker in a given sample was determined to be homozygous if the site was covered by 5 aligned reads from that individual samples and one allele had a frequency >0.9. The genotype for a given SNP marker in a given sample was determined to be heterozygous if the site was covered by 5 reads, at least 90% of the reads support the two most frequent alleles, at least two reads supported the two most frequent alleles, and both alleles had a frequency >0.2. Any cases which did not satisfy the conditions for either a homozygous or heterozygous SNP call were treated as missing data.

The initial set of SNPs was filtered to exclude any SNP site more than two alleles were identified, sites where only one genotype call was present, sites where more than 10% of samples with genotype calls heterozygous, sites where the minor allele was not identified in at least 5 samples, and sites where <20% of individuals had a genotype call for the site. These sets of filtered SNPs were used to calculate missing data rate (# of samples with missing data / total sample), heterozygosity (# of samples with heterozygous genotype calls / (# samples with homozygous genotype calls + # of samples with heterozygous genotype calls)) and minor allele frequency ((2×# of samples with homozygous minor allele genotype calls) + # of samples with heterozygous genotype calls) / 2 × total sample without missing sample). Then they were imputed using Beagle (Version: 16-06-2016). The filtered but unimputed and imputed SNP sets used in this paper have been uploaded to FigShare (https://doi.org/10.6084/m9.figshare.5566843.v1). Genetic markers with low MAFs (Minor Allele Frequency) are frequently removed prior to quantitative genetic analysis Tabangin *et al.* (2009). In downstream analysis, only SNPs with MAF larger than 0.05 were employed.

### Projecting Hybrid Genotypes

Hybrid genotypes for each possible combination of an A/B-line and an R-line were derived from genotypes of the corresponding parental inbred lines. If both parental lines were homozygous for the same allele at a given marker, the F1 progeny received the same genotype call at that marker. If the parental lines were homozygous for opposite alleles at a given marker, the F1 progeny received a heterozygous genotype call at that marker. If either parent was genotyped as heterozygous at a given marker was treated as having a genotype of (parent 1 genotype + parent 2 genotype)/2 on a scale of 0 to 2, where 0 is a genotype call of homozygous reference allele, and 2 is a genotype call of homozygous non-reference allele.

### Phenotype calculation

A linear mixed model was used to estimate the best linear unbiased prediction (BLUP) for the phenotypic traits of inbred and hybrid lines. In the model, genotype (G), location (L), genotype by location interaction (G×L), replication (R) and block (B) were treated as random effects.

In addition, the calculated variance of factors were used to estimate broad-sense heritability $\left(H_{2}\right)$ using the following formula (from Holland *et al.* (2003)):

$$H_{2}=\frac{V_{G}}{V_{G}+V_{G\times L}/N_{L}+V_{\epsilon }/\left(N_{R}\times N_{L}\right)}$$ (1)

where $N_{L}$ is the number of locations and $N_{R}$ is the number of replicates. $V_{G},$ $V_{G\times L}$ and $V_{\epsilon }$ represent the variance of the studied phenotypes controlled by genotype (G), interaction between genotype and environment (G×E) and residual factors.

In the analysis presented in Figure 2A, variance attributed to residual includes both $V_{\epsilon }$ as well as block and replicate variance. A customized R package (available at: https://jyanglab.github.io/g3tools/) was used to perform the above analyses.

The mid-parent heterosis for each trait was calculated from the BLUP values using the following formula:

$$\frac{Y_{hybrid}-\left(Y_{female}+Y_{male}\right)/2}{\left(Y_{female}+Y_{male}\right)/2}$$ (2)

### Genomic selection and cross-validation

BLUP values for lines with genotypic information – either direct genotyping for inbred lines or projected genotypes for hybrids – were used as training data for genomic prediction. For each approach described in results, predictions were conducted using the implementation of RR-BLUP (Ridge Regression Best Linear Unbiased Prediction) in the R-package rrBLUP Endelman (2011). The genomic prediction model was represented as:

$$y=\mu +\overset{k}{\underset{i=1}{\sum }}x_{i}g_{i}+e$$ (3)

where *y* is the matrix of BLUPs of all individuals, *μ* is the overall mean, k is the total number of SNPs, $x_{i}$ is the *i*th SNP genotype, $g_{i}$ is the effect for *i*th SNP and *e* is the residual.

For each trait, randomly selected subsets of SNPs ranging from 64 (2^6^) to 16,384 (2^14^) plus total projected hybrid SNPs were tested. For each subsampled set of SNPs, the individuals were divided into 5 groups, and five separate genomic prediction analyses were conducted, using four of the five groups as training data and the remaining group as testing data. The mean correlation coefficient across these five sub-predictions was treated as a single estimate of the accuracy of the prediction model for estimates of accuracy and standard deviation.

**Figure 1:**
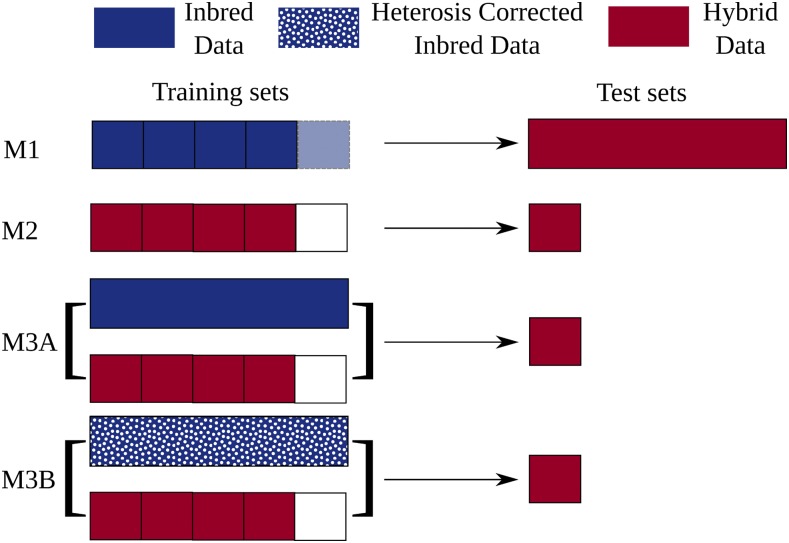
Four approaches taken to training and testing genomic prediction schemes. Scheme 1 (M1) uses different sets of 4/5s of the inbred phenotypic data to build a model which is tested by comparing predicted and measured traits for all hybrids. Scheme 2 (M2) is conventional fivefold cross validation, where the hybrids tested are divided into five equal parts, and the genomic estimated breeding values for hybrids in each are predicted using a model trained with the other four parts of the dataset. Scheme 3A (M3A), follows the same strategy outlined for M2, with the the training set extended to include the phenotypic and genotypic data for the inbred lines from M1. Scheme 3B (M3B) follows the same strategy as M3A but normalizes for the separate mean trait values of the inbred and hybrid populations prior to combining them into the training dataset.

The creation of the five individual sub-predictions varied somewhat across the four different prediction schemes described below (Figure 1). For each set of parameters with each scheme, a total of 20 sets of fivefold of cross-validation were performed. Thus, for each number of SNPs for each trait, a total of 20 × 5 = 100 sets of predictions were made. In scheme 1 (M1), the total set of genotyped and phenotyped inbreds was divided into five equal groups. Each sub-prediction used four of these five groups to predict phenotypic values for all genotyped and phenotyped hybrids. Scheme 2 (M2) utilized conventional five fold cross validation where the total set of genotyped and phenotyped hybrids was divided into five equal groups, and each sub-prediction used training data from four of the five groups to predict the remaining 20% of the data. The other two schemes utilized the same system as scheme 2, with the addition of all genotyped and phenotyped inbreds to the training dataset for all five subpredictions which either used BLUPs calculated across all individuals (M3A) or BLUPs calculated separately for inbred and hybrid populations (M3B).

To assess the accuracy of predictions for hybrids where one or more parents are completely unobserved, one B-line and one R-line were selected as “hold out” parents, and all hybrids which had either of these lines as a parent were excluded prior to the division of the remaining data into five groups. Each sub-prediction consisted of training the model using the hybrids four of these the five groups, and then predicting the genomic estimated breeding values of the hybrids with a “hold out” parent. Relative to the analysis without hold-out parents, prediction accuracy in this scenario decreased modestly and variance in prediction accuracy increased dramatically (Figure S2). Finally, all hybrids with genotype and genotype data were used to train a model that then produced genomic estimated breeding values for all 36,864 possible hybrid (Figure S3).

### Data availability

The authors affirm that all data necessary for confirming the conclusions of this article are represented fully within the article and its tables and figures. Supplemental material available at Figshare: doi: https://doi.org/10.6084/m9.figshare.5969230; doi: https://doi.org/10.6084/m9.figshare.5566843.

## Results

### Phenotype analysis

**Figure 2:**
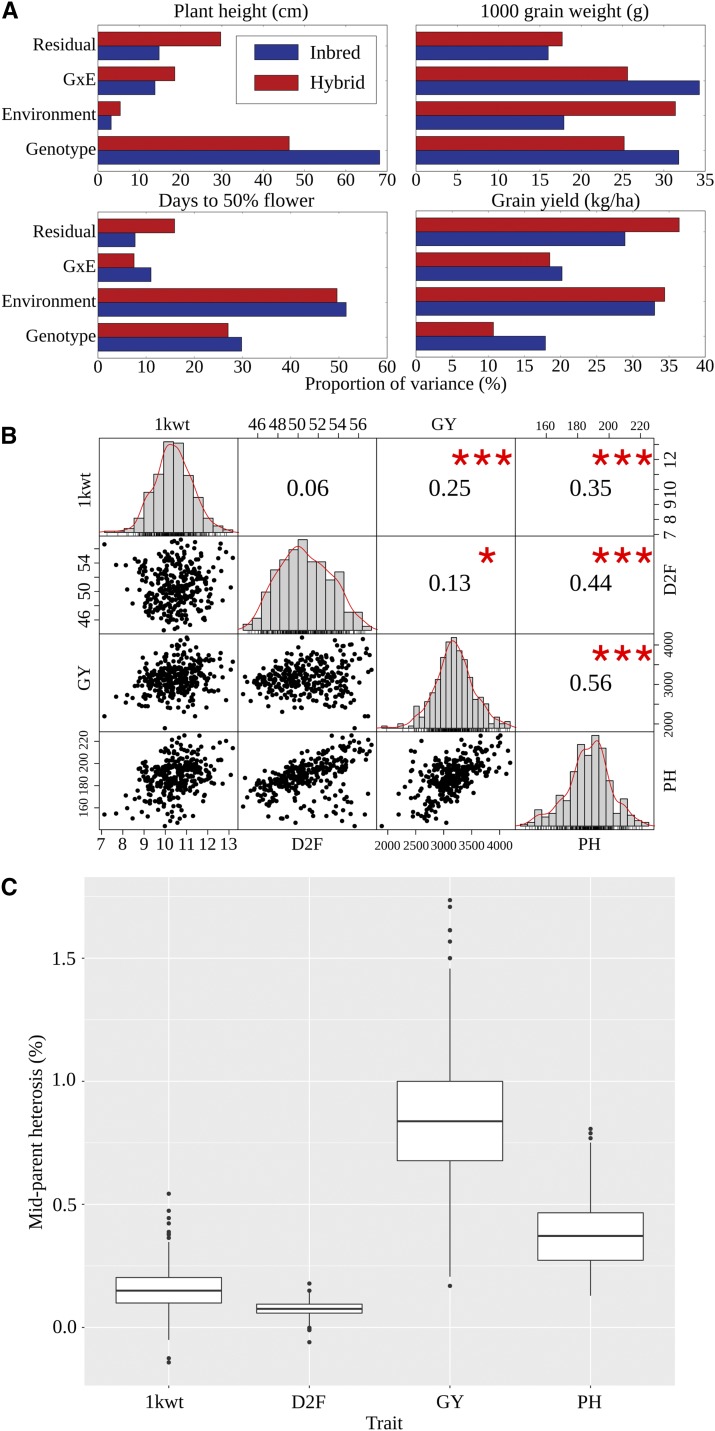
(A) Proportion of phenotypic variance explained by genotype, location (considered as a environmental factor), genotype by location (GxE) interaction for either inbred pearl millet lines or hybrid pearl millet lines. (B) Phenotype investigation of four studied traits in pearl millet population. *** p value of the significance of this correlation is ≤ 0.001, ** p value of the significance of this correlation is ≤ 0.01 and * p value of the significance of this correlation is ≤ 0.05; (C) Distribution of observed mid-parent heterosis for each of the four traits scored in this study.

Phenotypic variance was partitioned into four components: genotype (G), environment (E), interaction between genotype and environment (G×E), residual (R). For each trait, the pattern of relative contribution of each of these factors was roughly similar between inbred and hybrid pearl millet populations (Figure 2A). Plant height was the trait with the greatest proportion of variance explained by purely genetic factors, while flowering time had the great proportion of variance explained by environments. As expected, grain yield had the highest residual value, making this critical trait the most difficult to predict accurately using quantitative genetic models. Broad sense heritability – *i.e.*, the proportion of total variance in trait values explained by genetic factors – for grain yield, plant height, flowering time and 1000-grain weight were estimated to be 0.60, 0.86, 0.88 and 0.74 respectively for pearl millet hybrids and 0.72, 0.92, 0.88 and 0.74 for inbreds. However, caution should be used in interpreting differences between the inbred and hybrid heritability values, given the large difference in the number of individuals between the two populations. BLUP values were calculated for these four traits (see methods). Each trait exhibited an approximately normal distribution (Figure 2B). Heterosis can be defined in different ways but the definition employed here is mid-parent heterosis which is the degree to which the measured trait values of hybrids tend to exceed the average of measured values for the same trait in both parents. Mid-parent heterosis was observed for all four traits with median values of 8% (flowering time), 15% (1000-grain weight), 37% (plant height) and 84% (grain yield) (Figure 2C). Note that the direct of effect for heterosis was reversed for flowering time. This is consistent with studies in maize which indicate that hybrid tend to flower earlier than their parents Dickert and Tracy (2002).

### Characteristics of the tGBS and RAD-seq datasets

A total of 4,550 million barcoded RAD-seq reads were generated on an Illumina HiSeq 2000, for an average of 12.2 million reads per sample. tGBS libraries were sequenced using seven Ion Proton runs, generating a total of 810 million raw reads included 584 million of barcoded reads, for an average of 2.1 million reads per sample.

**Figure 3:**
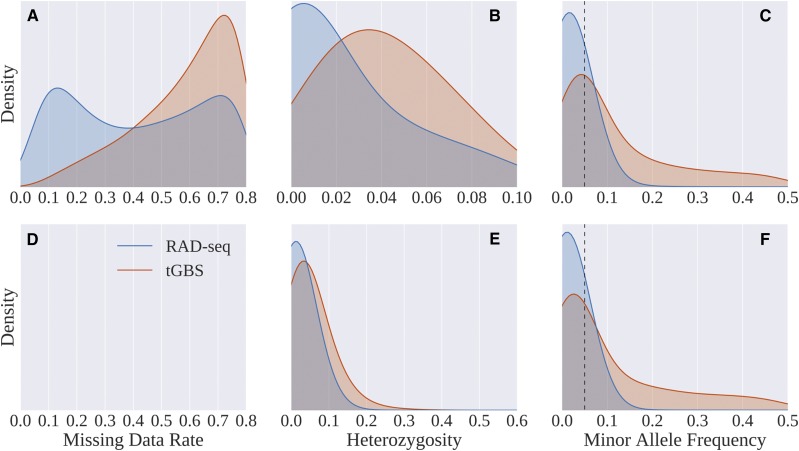
Distribution of missing data rates (A, D), heterozygosity (B, E), and minor allele frequency (C, F) for SNPs identified and scored in either the RAD-seq or tGBS dataset. A-C summarize raw SNP data prior to imputation. D-F show densities for the same characteristics subsequent to imputation. However, no missing sites were left after imputation, hence panel D is blank. Dashed line in C & F indicates the cut off of MAF = 0.05 for SNPs which were utilized in downstream genomic prediction.

After aligning to the pearl millet reference genome and quality filtering (See Methods), 649,067 polymorphic SNPs were identified using RAD-seq data and 73,291 SNPs were identified using tGBS data. As expected given the different subsets of the genome targeted by these two technologies Miller *et al.* (2007); Ott *et al.* (2017), there was only minimal overlap between the two methods with only 439 SNPs identified and scored by both technologies. The missing data rates for RAD-seq genotypes exhibited a bimodal distribution while tGBS genotypes exhibited a unimodal distribution skewed toward high missing data rates. RAD-seq genotyping was much less likely to genotype sites as heterozygous, which may reflect a difference in the technologies, or may be explained by the observation that many SNPs identified by RAD-seq had low minor allele frequencies, while tGBS SNPs, tended to have higher minor allele frequencies (Figure 3). A more detailed comparison of the outcomes of RAD-seq and tGBS genotyping is provided in Table 1. Downstream analyses utilized only those SNPs with MAF >0.05 from each dataset (15,306 RAD-seq SNPs and 32,463 tGBS SNPs).

### Evaluating the accuracy of genomic prediction

The ability of genomic prediction using projected hybrid genotypes from both genotyping methods was then assessed for each phenotype using cross validation. For each tested set of SNPs, 20 random rounds of fivefold cross validation were performed. The median correlation coefficient of 1000-grain weight, days to flowering time, grain yield and plant height using all available SNPs was 0.73, 0.89, 0.51 and 0.72 for RAD-seq and 0.74, 0.87, 0.48 and 0.73 for tGBS (Figure S1). The differences in prediction accuracies observed for the two methods, either utilizing random sub-sampling of equal numbers of SNPs for each dataset or all SNPs obtained using each genotyping method were not statistically significant (student’s *t*-test).

### Comparison of prediction models

**Figure 4:**
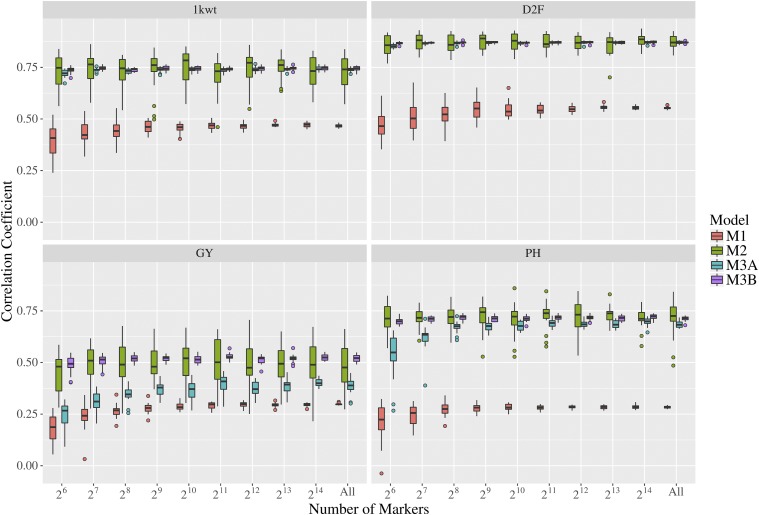
Prediction accuracy for each of four phenotypes scored in this pearl millet population employing the four schemes outlined in Figure 1 using tGBS SNP calls. Scheme 3A (M3A) employed absolute predicted trait values for inbreds and hybrids to train a genomic prediction model, while scheme 3B (M3B) employed predicted trait data for inbreds and hybrids calculated relative to the separate mean trait values for inbred and hybrid lines.

**Figure 5:**
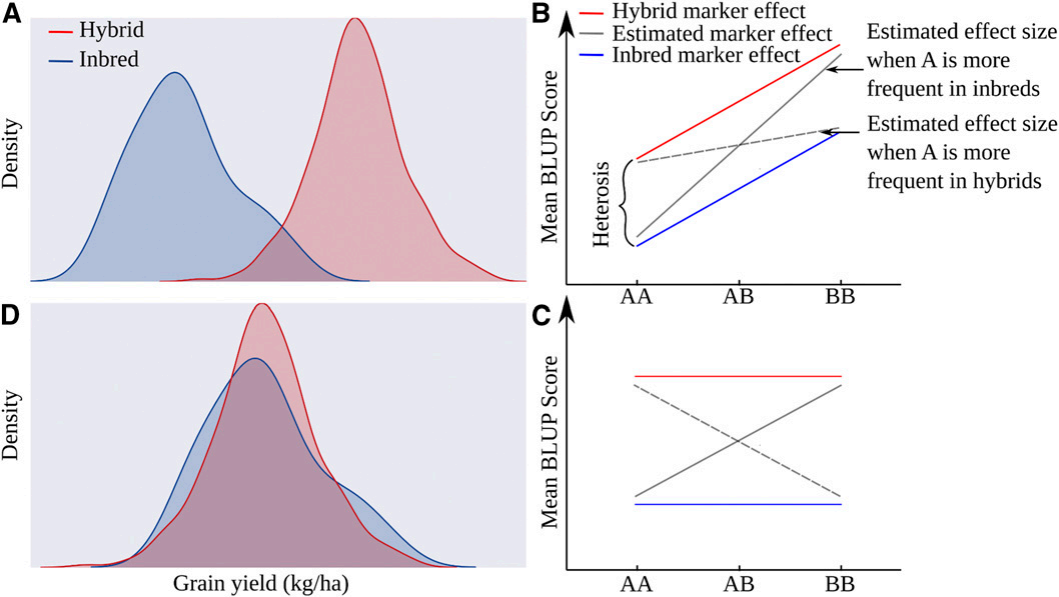
A proposed model for the decrease in genomic prediction accurary for high heterosis traits when inbred individuals are introduced into training populations. A) Distribution of BLUP scores for yield for populations of hybrid and inbred individuals based on a combined BLUP analysis. B) Distribution of scores for a hypothetical marker having an equally large effect size in inbred and hybrid individuals. When allele frequencies differ between these populations, and the ratio of hybrid to inbred individuals may vary between the groups of individuals with genotype AA or with genotype BB. C) Distribution of scores for a hypothetical marker with no effect on trait value. D) Distribution of BLUP scores for yield for populations of hybrid and inbred individuals based on a separate BLUP analysis for hybrid and inbred individuals.

Four different approaches (see Methods) to genomic prediction were evaluated to test whether inbred phenotypic data can add value to genomic prediction as part of a hybrid breeding program. Scheme M1, which used trait trait from inbreds to predict genomic estimated breeding values of hybrids performed the worst of all four approaches for all four phenotypic traits tested. Notably, the rank of traits by mid-parent heterosis had a perfect negative correlation with the rank of the traits by phenotypic prediction accuracy using inbred parent training data. Scheme 2 (M2) produced a significant increase (*P* < 0.05) in accuracy relative to the scheme 1 (M1) for all four phenotypes, although, consistent with its high residual values when fitting the original BLUP, the accuracy of prediction for yield was the lowest of the four phenotypes (Figure 4). Scheme 3A (M3A), which merged phenotypes from both inbred parents and hybrid trials to predict hybrid trials performed equivalently to scheme 2 (M2) for 1000-grain weight (1kwt) and flowering time (D2F), the two traits with the lowest degree of mid-parent heterosis. However, for traits with significant amounts of mid-parent heterosis (grain yield and plant height), adding inbred parent phenotype data to the prediction model either provided no benefit (plant height, *P* = 0.17) or significant *decreases* in prediction accuracy (grain yield, *P* = 0.09e-2) compared to a purely hybrid phenotype data training set. Scheme 3B (M3B), which, instead of employing absolute trait values for inbreds and hybrids, summarized phenotypes as the differences between the predicted trait value for a given inbred or hybrid line and the mean trait value for either all hybrids or all inbreds (Figure 5D), performed approximately equal to, or sometimes marginally better than M2 (hybrid only scheme). An additional 1,000 permutations of fivefold cross validation were conducted for scheme 2 (M2) and scheme 3B (M3B) using the “All SNPs” dataset. The increase in prediction accuracy in M3B relative to M2 was statistically significant for two out of four traits tested: flowering time (*P* = 6.00e-4), and grain yield (*P* = 5.03e-9).

Increases in prediction accuracy coming from increasing numbers of markers tended to plateau at smaller total marker numbers for scheme 1 (M1 which is inbred only) than for scheme 2 (M2 which is hybrid only), with scheme 3 (M3 which is inbreds plus hybrids) was intermediate between the two. However, even 64 (2^6^) random SNPs provided significant (*P* < 0.05) predictive ability for all traits and all schemes tested. The relatively small set of inbred parents used to create the set of hybrids tested as part of this analysis may have resulted in inflated apparent prediction accuracies for each trait. When using a hold-two-parents out approach to segregating hybrids with common parentage between the training and testing datasets (see Methods), accuracy was lower and standard deviations of prediction accuracy were higher (Figure S2), indicating that our estimates of prediction accuracy are likely to be optimistic relative to the ability to predictions for hybrids where one or both parents have not previously served as parents for tested hybrids.

Finally, grain yield and time to flowering were predicted for every possible F1 hybrid between a genotyped A/B-line and a genotyped R-line in the dataset. Within this prediction space, the highest yielding potential hybrids tend to be associated with somewhat longer flowering times defining a production possibility frontier for the trade off between growing season length and yield among the pearl millet hybrids which could be generated using the inbred germplasm genotyped as part of this study (Figure S3).

## Discussion

Here we found that the naive integration of trait data collected from inbred lines into genomic prediction for a hybrid breeding program can actually reduce prediction accuracy, particularly for traits exhibiting significant heterosis. However, controlling for the effect of heterosis *per se* by calculating BLUPs separated for inbred and hybrid lines eliminated this negative impact on prediction accuracy and could in fact increase prediction accuracy for some traits relative to predicting using data from hybrid lines alone. In addition we found prediction accuracy was equivalent for SNPs generated through either RAD-seq or tGBS. Given the greater number of high MAF (>0.05) SNPs generated per million reads with tGBS, this methodology is likely to be more cost effective in the context of many genomic selection based breeding programs. In addition, the more rapid turn-around time enabled by Ion Proton sequencing (∼4 hr) relative to Illumina HiSeq 2000 (∼8 days for 2x100 sequencing) increases the feasibility the utilizing genomic prediction to guide real time breeding decisions. However, it should also be noted that there is nothing inherent about either the RAD-seq or tGBS protocol which prevents the adaptation of either protocol to sequencing using either instrument.

Longer growing seasons will generally – in the absence of constraints from temperature, or resources abundance – result in more total fixed carbon Dohleman and Long (2009). Whether this increase in carbon fixation results in an increase in yield depends, among other factors on harvest index, the partitioning of carbon between vegetative and reproductive development. In our data, grain yield displayed only weak correlations with flowering time (Figure 2B). Grain yield and plant height show a strong positive correlation with each other which is not what would be expected based on models of carbon partitioning. One potential explanation is that, in small plot trials with substantial height variation among accessions, tall plots can shade shorter plots if the experimental design is not blocked by height. This shading effect produces an apparent yield penalty for short accessions which does not translate to larger scale yield trials or commercial production. However, there may also be significant room to improve the yield and resource use efficiency of pearl millet through selection for improved harvest index. A second explanation is loci responsible for tolerance of heat and or drought stress are segregating in the population, sensitive genotypes are likely to exhibit both stunted growth and low grain yields, as pearl millet is grown in marginal soils and high levels of abiotic stress (high temperatures and water constraint).

As described above, we found that the naive incorporation of inbred genotype and trait information into training datasets decreased prediction accuacy for high heterosis traits. In Figure 5, we propose a model to explain this finding. When BLUP values for a trait with a high degree of heterosis are calculated in a population containing a mix of inbred and hybrid individuals, most inbreds will be assigned negative BLUP scores and most hybrids postive BLUP scores (Figure 5A). Distributions of allele frequencies will vary between inbred and hybrid populations. As a result, inbred individuals may be relatively more common among the population of individuals with AA or BB genotypes. Alleles more common in inbred individuals relative to hybrid individuals will tend to be assigned a more negative or less positive effect value by a genomic prediction model trained with a mixed hybrid/inbred population than by a genomic prediction model trained on a purely hybrid or purely inbred population (Figure 5B Figure 5C). Calculating BLUPs separately for inbred and hybrid individuals removes this source of bias in the training data by centering the distributions of both inbred and hybrid individuals (Figure 5D).

Consistent with earlier studies in maize, we found that inbred trait values alone were poor predictors of hybrid performance, particularly for yield e Gama and Hallauer (1977); Smith (1986), although the prediction values in scheme 1 (M1), trained only on inbred data were at least statistically significantly greater than zero (*P* < 0.05). Here we found that when using a conventional additive genomic prediction model (RR-BLUP), traits with higher median heterosis (grain yield and plant height) experienced a decrease in prediction accuracy when inbred data were naively incorporated into the training dataset (M3A). Segregating BLUP means for inbreds and hybrids (M3B) statistically significantly moderately enhanced prediction accuracy for grain yield (*P* = 5.03e-9) and flowering time (*P* = 6.00e-4), compared to a scheme which excluded phenotypic data from inbred parents (M2). While statistically significant, the absolute values of the increases in prediction accuracy are moderate: flowering time (M2: 0.87, M3B: 0.87) and grain yield (M2: 0.50, M3B: 0.52). Yield was both the most difficult trait to predict, and the trait where the inclusion of inbred trait data provided the largest increase in prediction accuracy. In this study the number of inbred lines for which genotypic and phenotypic data were available was quite small. Even in scheme 3B, inbred lines made up less than 15% of the training dataset. Given that extensive inbred trait datasets exist for pearl millet, it may be that the incorporation of genotypic and phenotypic data from larger numbers of inbred lines would produce a larger absolute increase in prediction accuracy. As inbred lines must be grown prior to the production of hybrid seed, the collection of trait data from these lines comes at relatively low cost, and may have additional value when integrated into training datasets which also include genotypic and phenotypic data from a sample of hybrid lines.

Even small numbers of selected SNPs can achieve relatively high prediction accuracy in this pearl millet population. The implementation of a hybrid GS/GP guided pearl millet breeding program has the potential to significantly improve the efficiency of breeding efforts (Figure 4). However, it must be noted that in our training set the high representation of haplotypes drawn from 33 common parental lines produces close relationships between sub-sampled training and testing populations, and this could also be a reason to explain why a smaller number set of SNPs can reach plateaus in accuracy for genomic prediction of some studied traits. As a result, our estimates of model prediction accuracy are likely inflated relative to predictions on unrelated populations Isidro *et al.* (2015). To expand the applicability of this genomic prediction model to a wider pearl millet genomic selection assisted breeding program, it will be necessary to incorporate data from a hybrids derived from a broader genetic basis.
